# Rbfox2 dissociation from stress granules suppresses cancer progression

**DOI:** 10.1038/s12276-019-0246-y

**Published:** 2019-04-26

**Authors:** Sunkyung Choi, Moa Sa, Namjoon Cho, Kee K. Kim, Su-Hyung Park

**Affiliations:** 1 Department of Biochemistry, College of Natural Sciences, Chungnam National University, Daejeon, 34134 Republic of Korea; 2 Biomedical Science and Engineering Interdisciplinary Program, Korea Advanced Institute of Science and Technology, Daejeon, 34141 Republic of Korea; 3 Graduate School of Medical Science and Engineering, Korea Advanced Institute of Science and Technology, Daejeon, 34141 Republic of Korea

**Keywords:** Targeted therapies, Transcriptomics, Chemotherapy

## Abstract

Stress granules (SGs) are stalled translation initiation complexes comprising untranslated mRNAs and RNA-binding proteins (RBPs). RBP fox-1 homolog 2 (Rbfox2), a component of SGs, binds to *retinoblastoma 1* (*RB1*) mRNA, which is closely related to cancer progression; however, the role of Rbfox2 in cancer progression remains largely unknown. In this study, we confirmed that Rbfox2, which is present in the nucleus as a splicing regulator, localizes to the cytoplasm of human colon cancer tissues and that induction of Rbfox2 dissociation from SGs by resveratrol treatment inhibits cancer progression. We also observed that Rbfox2 in SGs inhibited RB1 protein expression and promoted cell cycle progression. Additionally, resveratrol treatment inhibited SG-mediated Rbfox2 localization, further inhibiting RB1 protein expression, and inhibited specific Rbfox2 localization to the cytoplasm in melanoma B16-F10 cells, thereby effectively inhibiting metastasis and tumor growth ability. These results indicate that Rbfox2 dissociation from SGs attenuates cancer progression and offer insight into the mechanism associated with Rbfox2 dissociation, thereby marking Rbfox2 as a potential candidate target for cancer therapy.

## Cancer: Interfering with RNA processing to prevent tumor growth

Resveratrol, an antioxidant found in red grapes, slows cancer progression by interfering with the localization and function of the RNA-binding protein Rbfox2. A study led by Kee Kim at Chungnam National University and Su-Hyung Park at Korea Advanced Institute of Science and Technology, Daejeon, Republic of Korea, showed that in human colon cancer cells Rbfox2 is located in the cytoplasm where it promotes cell proliferation by blocking the assembly of the tumor suppressor protein RB1. Treatment with resveratrol prevented the migration of Rbfox2 from the nucleus to the cytoplasm and significantly reduced tumor growth in a mouse model of melanoma. This study not only sheds light on the protective effects of resveratrol but also suggests that Rbfox2 could be a potential target for the development of new anticancer drugs.

## Introduction

Nascent mRNAs bind to RNA-binding proteins (RBPs) to form a complex of ribonucleoprotein particles (RNPs)^1^. In the RNP complex, RBPs play an important role in gene expression by determining mRNA decay/stabilization, subcellular localization, and translation rates^2–4^. RNP granules formed by the aggregation of RBPs and RNAs form non-membrane-bound cellular compartments. Depending on their composition, subcellular localization, response to stimuli, and function, RNP granules can contain processing bodies, stress granules (SGs), neuronal granules, and nuclear paraspeckles^5–7^. SGs include translation initiation factors, 40 S ribosomal subunits, and nontranslatable mRNAs that rapidly associate with and assemble various RBPs to promote their dense aggregation in the cytoplasm^8,9^. SG formation is generally triggered by unfavorable environmental conditions, such as heat-shock, chemical exposure, oxidative stress, and aging, to protect stressed cells^9,10^. SG formation occurs through phosphorylation of eukaryotic initiator factor 2 (eIF2) and is a critical process for translation reprogramming, mRNA degradation, and untranslated mRNA storage^9–13^.

Recent studies have reported that SGs contain signaling and catalytic proteins as well as transcripts and translation components, suggesting that rearrangement of RNP complexes by SG assembly and disassembly can critically affect cell metabolism and survival^1^. Indeed, uncontrolled formation of SGs is associated with the pathogenesis of various diseases, including neurodegenerative, inflammatory, and infectious diseases^14,15^. T-cell intracytoplasmic antigen-1 (TIA-1)-related protein, tristetraprolin, and Ras-GTPase-activating protein SH3-domain-binding protein 1/2 (G3BP1/2) are associated with pathologic lesions of neurodegenerative diseases and are closely associated with SGs^16,17^.

RBPs regulate the post-transcriptional processing of many cancer-related genes, including tumor suppressors and oncoproteins^18,19^. Abnormal expression of eIF4E induces malignant transformation in mouse and rat fibroblasts^20^. Additionally, the human antigen R (HuR) protein binds to adenine- and uridine-rich elements (AREs) in the untranslated regions (UTRs) of cancer-associated mRNAs, including proto-oncogene, growth factor, cytokine and invasion factor mRNAs, and regulates the stability of the target transcripts^21^. Although some RBPs are associated with tumorigenesis, neurodegenerative diseases caused by SGs with localized RBPs have been the primary research focus to date, and the role of SGs in cancer remains unclear.

We have previously shown that RBP fox-1 homolog 2 (Rbfox2), a typical RBP that regulates alternative pre-mRNA splicing in the nucleus, binds to the mRNA of cell cycle-related genes, including *retinoblastoma 1* (*RB1*), in SGs^22^. Because RB1 acts as an important and regulates cell growth and division, its expression is a primary factor associated with many cancers, including retinoblastoma^23^. Therefore, in this study, we confirmed that Rbfox2 dissociation from *RB1* mRNA associated with SGs following resveratrol treatment inhibited cancer progression. Additionally, we observed that Rbfox2 localized to the cytoplasm of human colon cancer cells and that RB1 protein levels were very low in human colon cancer tissue. Furthermore, we verified that Rbfox2 located in SGs stimulated cell cycle progression and cell proliferation by inhibiting RB1 translation. Moreover, the results showed that resveratrol treatment inhibited SG-mediated Rbfox2 localization and attenuated lung metastasis in a mouse model.

## Materials and methods

### In vivo tumorigenesis model

Six-week-old male C57BL/6 J mice were obtained from Daehan Biolink (Eumseong-gun, Korea) and were maintained in an air-conditioned facility at 25 ± 2 °C with a relative humidity ranging from 40 to 70% and a 12-h light/dark cycle. B16-F10 melanoma cells (5 × 10^5^) were subcutaneously injected into C57BL/6 J mice. Mice were intraperitoneally (IP) injected with resveratrol (1 mg/kg) or vehicle once daily beginning on the day prior to the injection of the B16-F10 cells. The dimensions of each tumor were measured on two days using Vernier calipers, and the tumor volume was estimated with the following formula: tumor volume = π/6 × (major axis) × (minor axis)^2^. The mice were sacrificed at the end of the experiment, and the tumors were isolated.

Mice were engrafted by intravenous injection in the tail vein of 2 × 10^5^ B16-F10 cells to initiate lung metastasis. The mice were also intraperitoneally injected with 1 mg/kg resveratrol or vehicle once daily beginning on the day prior to injection of B16-F10 cells. After treatment, the mice were sacrificed on day 14, and the lungs were dissected. All animal-related procedures were approved by the Institutional Committee for Animal Care and Usage, KAIST (Daejeon, Korea), and were performed according to the institutional guidelines.

### Cell culture

Human cervical HeLa cells (ATCC, Manassas, VA, USA) and *Mus musculus* skin melanoma B16-F10 cells (ATCC) were cultured in Dulbecco’s modified Eagle medium (WELGENE, Gyeongsangbuk-do, Korea) containing 10% fetal bovine serum (Gibco; Thermo Fisher Scientific, Waltham, MA, USA) and 1% penicillin/streptomycin (WELGENE) at 37 °C in a humidified atmosphere containing 5% CO_2_.

### Small interfering (si) RNA transfection

The sense sequence of siRNA against human Rbfox2 (5′-GGGAUUCGGGUUCGUAACU-3′) and a nontargeting control siRNA were obtained from Dharmacon (Lafayette, CO, USA). Transfection was performed using Amaxa Nucleofector (Lonza, Basel, Switzerland) according to the manufacturer’s instructions. The transfected cells were analyzed after 36 h.

### Flow cytometry to measure cell cycle progression

Cells were harvested, fixed with 70% ethanol at 4 °C overnight, and stained with 500 μg/mL propidium iodide solution containing 50 μg/mL RNase at 37 °C for 1 h. The treated cells were subjected to flow cytometry using a FACSCalibur system (Becton-Dickinson, Franklin Lakes, NJ, USA) to analyze cell cycle distribution. The data were quantified using FlowJo software (FlowJo, LLC, Ashland, OR, USA).

### Immunoprecipitation and immunoblot analysis

Whole cells were lysed in mammalian protein extraction reagent (M-PER; Thermo Fisher Scientific) with a protease inhibitor cocktail (Roche Applied Science, Schlieren, Switzerland). For immunoprecipitation analysis, HeLa cell lysates (1 mg of protein) were incubated with anti-Rbfox2 antibody (Bethyl Laboratories, Montgomery, TX, USA) for 4 h at 4 °C. Furthermore, Dynabeads Protein G (Invitrogen, Carlsbad, CA, USA) were added to the lysate and antibody mixture, and the mixture was incubated for 2 h. The immune complexes were washed with M-PER buffer six times. For DNase treatment, the beads coated with the lysate and antibody mixture were incubated in 200 U/mL Turbo DNase (Thermo Fisher Scientific) at 25 °C for 10 min and washed again with M-PER buffer. The proteins were eluted by boiling in Laemmli sample buffer (Bio-Rad, Hercules, CA, USA).

For immunoblot analysis, proteins were separated on 4–20% NuPAGE Bis-Tris gels (Thermo Fisher Scientific) and transferred to nitrocellulose membranes, which were subsequently blocked with 5% skimmed milk and incubated overnight at 4 °C with primary antibodies. After washing, the blots were incubated with peroxidase-conjugated secondary antibodies (Abcam, Cambridge, UK) for 1 h at room temperature. The proteins were detected using enhanced chemiluminescence reagents (SuperSignal; Thermo Fisher Scientific). The primary antibodies used in this study were anti-AMPKα1/2 (Santa Cruz Biotechnology, Dallas, TX, USA), anti-phospho-AMPKα Thr172 (Cell Signaling Technology, Danvers, MA, USA), anti-glyceraldehyde 3-phosphate dehydrogenase (GAPDH; Meridian Life Science, Memphis, TN, USA), anti-RB1 (Santa Cruz Biotechnology), anti-Rbfox2 (Bethyl Laboratories), anti-ubiquitin (Cell Signaling Technology), and anti-phospho-(Ser/Thr) AMPK substrate (Cell Signaling Technology).

### Immunofluorescence microscopy

Human and mouse tissue samples were fixed in 4% (w/v) paraformaldehyde for at least 24 h, and then immersed overnight in 30% (w/v) sucrose solution before being embedded in Tissue-Tek optimum cutting temperature compound (Sakura Finetek, Torrance, CA, USA) for cryosectioning. The frozen tissues were cut into 12-μm-thick sections and stored at −80 °C. HeLa cells were fixed in 4% paraformaldehyde for 10 min and permeabilized with 0.5% (v/v) Triton X-100 in phosphate-buffered saline (PBS) for 15 min.

All samples were blocked with 5% goat serum and 0.1% bovine serum albumin for 1 h and incubated overnight at 4 °C with primary antibodies. After washing the slides, Alexa Fluor 488- and 532-conjugated goat antibodies against rabbit and mouse IgG (Thermo Fisher Scientific), respectively, were used as secondary antibodies, and nuclei were stained with 4′,6-diamidino-2-phenylindole (DAPI; Thermo Fisher Scientific). The slides were then washed five times with PBS for 10 min each and mounted with ProLong Gold antifade mounting medium (Thermo Fisher Scientific). Images were acquired using a Zeiss LSM 510 Meta confocal laser-scanning microscope (Carl Zeiss, Oberkochen, Germany). The primary antibodies used in this study included rabbit polyclonal anti-Rbfox2 (Bethyl Laboratories), mouse monoclonal anti-G3BP1 (Santa Cruz Biotechnology), mouse monoclonal anti-E-cadherin (Cell Signaling Technology), and mouse monoclonal anti-tubulin (Sigma-Aldrich, St. Louis, MO, USA).

### RBP immunoprecipitation (RIP) polymerase chain reaction (PCR)

Cells were lysed with RIP buffer [150 mM KCl, 25 mM Tris (pH 7.4), 5 mM EDTA, 0.5 mM DTT, 0.5% NP40, and 100 U/mL RNase inhibitor (Enzynomics, Daejeon, Korea)] supplemented with a protease inhibitor cocktail (Roche Applied Science). Cell lysates containing 600 μg of protein were incubated with anti-Rbfox2 antibody for 4 h at 4 °C. The lysate was centrifuged at 10,000 × *g* for 10 min, and the supernatant was precleared by interaction with Dynabeads (Protein G; Invitrogen, Carlsbad, CA, USA) for 2 h at 4 °C with constant shaking. The Dynabead Protein G/antibody/protein complexes were washed six times with RIP buffer, and total RNA was extracted using a Direct-zol RNA MiniPrep kit (Zymo Research, Irvine, CA, USA). The RNA was then reverse transcribed by M-MLV reverse transcriptase (Promega, Madison, WI, USA) with random hexamers. The primers for reverse transcription (RT)-PCR were as follows: RB1-F (5′-ACAGATTTGTCCTTCCCGTG-3′) and RB1-R (5′-CCATGATTCGATGCTCACAT-3′). The RT-PCR end products were visualized by 1.8% agarose gel electrophoresis.

### Reverse phase protein microarray (RPMA)

RPMAs for human colon cancer and normal tissue were purchased from Protein Biotechnologies (Ramona, CA, USA), and assays were performed according to the manufacturer’s instructions. Briefly, the slides were washed with distilled water to wet the nitrocellulose membranes. After the slides were washed, they were rinsed twice with PBS containing 0.1% Tween-20. The slides were blocked using ZeptoBlock Protein Microarray Blocking Buffer in a ZeptoSeal^TM^ incubation chamber for 1 h at room temperature. The slides were then incubated overnight at 4 °C with anti-RB1 or anti-Rbfox2 antibody in ZeptoBlock^TM^ and washed six times with PBS containing 0.01% Tween-20. The binding of horseradish peroxidase-conjugated secondary antibody was detected using the SuperSignal system. Images were captured by scanning the slides on a high-resolution digital scanner, and data analysis was performed. The RPMA samples were confirmed as having been collected under strict institutional review board-approved guidelines/protocols with informed consent. All collected human tissue proteins were maintained under strict confidentiality and in accordance with the appropriate laws that protect the confidentiality of personal information.

### Data and statistical analysis

The data and statistical analysis methods complied with recommendations for experimental design and analysis in pharmacology^24^. The data are presented as the mean ± SEM. Statistical analysis was performed using GraphPad Prism 5 (Prism, La Jolla, USA). *P* < 0.05 was considered to indicate statistical significance.

### Materials

Sodium arsenite (arsenite), resveratrol, 5-aminoimidazole-4-carboxamide ribonucleotide (AICAR), and compound C were purchased from Sigma-Aldrich. The proteasome inhibitor MG132 (carbobenzoxy-L-leucyl-L-leucyl-L-leucinal) was purchased from Calbiochem (Darmstadt, Germany).

## Results

### Rbfox2 predominantly localizes to the cytoplasm in colon cancer cells

**Fig. 1:**
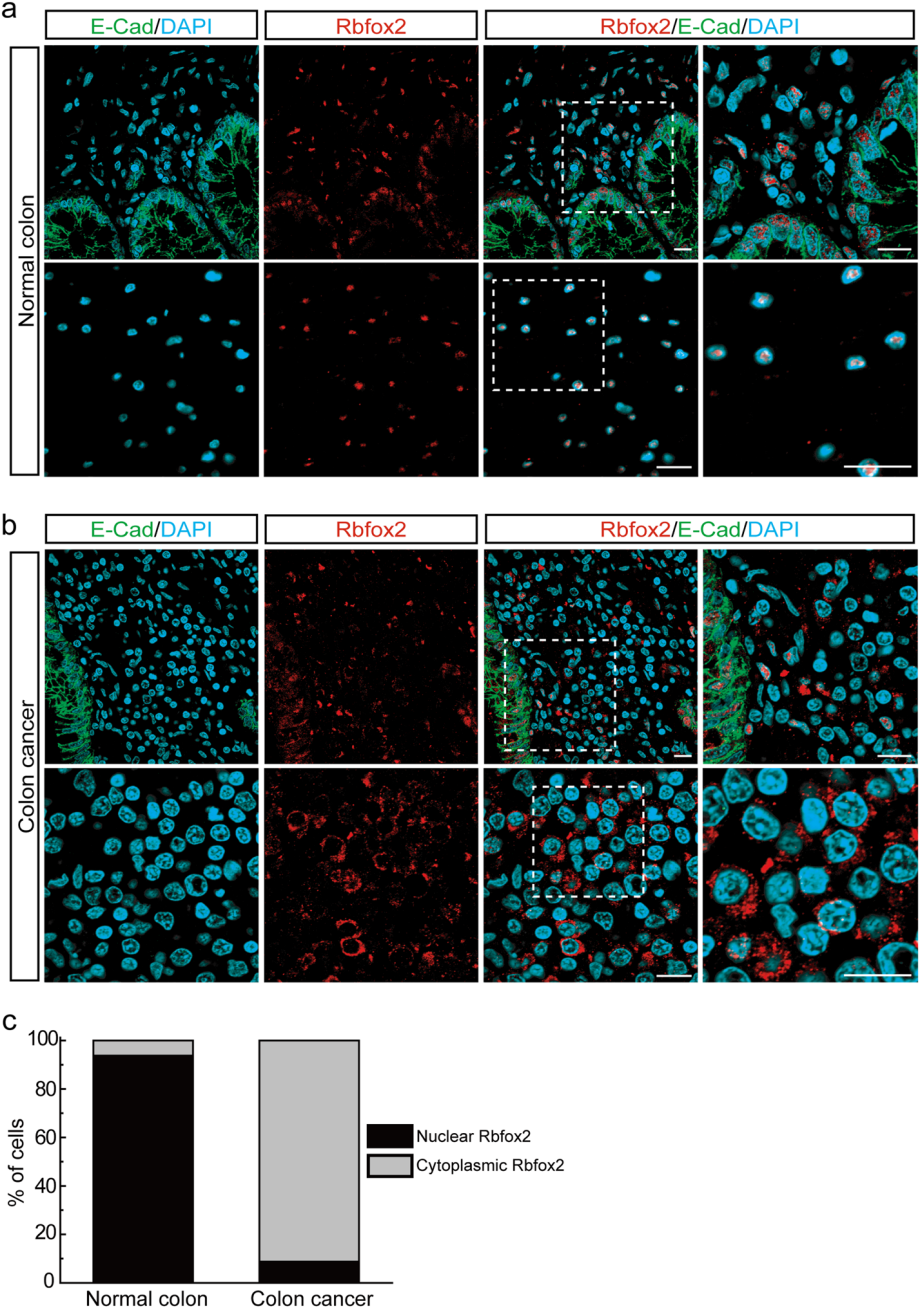
Rbfox2 exhibits different subcellular localization between normal and cancer cells. Confocal immunofluorescence microscopy of cryosections from (**a**) human normal colon tissue and (**b**) colon cancer tissue labeled with E-cadherin (green) and anti-Rbfox2 (red). DAPI (blue) staining represents the nuclei. The white-boxed region is magnified (*right*). Scale bars, 10 μm. **c** Quantification of Rbfox2 localization in human normal colon and colon cancer tissue

Rbfox2 localizes to cytoplasmic granules under stress conditions;^22^ however, the tumor microenvironment differs markedly from that of normal tissues with regard to stress-inducing conditions. To investigate Rbfox2 localization in tumor tissues, we examined the subcellular localization of Rbfox2 by immunofluorescence microscopy of cells derived from colon tissue. To define colon cancer tissue compared with normal colon tissue, we used two features: E-cadherin expression and nuclear size. E-cadherin is a cancer marker known to be significantly less expressed in colon cancer tissue compared with normal colon tissue^25^. As expected, the expression level of E-cadherin in normal colon tissue was higher than its expression level in colon cancer tissue (Fig. 1). In addition, nuclear size differs between normal and cancerous cells^26,27^. We found that nuclear size was larger in colon cancer tissue than in normal colon tissue (Fig. 1). Although we observed nuclear localization of Rbfox2 in cells from normal colon tissue (Fig. 1a), we observed strong cytoplasmic staining of Rbfox2 in different regions of cells from colon cancer tissue (Fig. 1b). The localization of Rbfox2 in normal colon and colon cancer tissues was quantified (Fig. 1c). The results suggest that the subcellular localization of Rbfox2 to the cytoplasm is cancer cell-specific.

### Rbfox2 in SGs promotes cell cycle progression

**Fig. 2:**
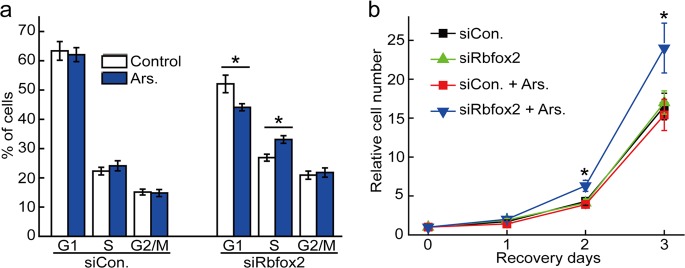
Rbfox2 regulates cell cycle progression. **a**, **b** HeLa cells were transfected with siRNA against Rbfox2 (siRbfox2) or with nontargeting control siRNA (siCon.). After 36 h, the cells were treated with 500 μM arsenite for 40 min (Ars.). **a** After 24 h of incubation, the cells were subjected to cell cycle analysis using FACS. The graph shows the percentage of the cell population at the G1, S, and G2/M phases. The bars represent the mean ± SEM of three independent experiments (*n* = 3). *p < 0.05 compared with the corresponding controls. **b** For recovery, the cells were incubated in complete medium for 1, 2, and 3 days, and cell counts were obtained using a hemocytometer. The bars represent the mean ± SEM of three independent experiments (*n* = 3). *p < 0.05 compared with the corresponding controls

Cytoplasmic Rbfox2 regulates RB1 expression under stress conditions^22^. RB1 is a negative cell cycle regulator involved in the G1/S cell cycle checkpoint and was the first tumor-suppressor gene identified as dysfunctional in several major cancers. Given that Rbfox2 binds to *RB1* mRNA and that this interaction influences RB1 protein expression under stress conditions, we investigated the role of Rbfox2 binding to *RB1* mRNA in cell cycle progression. First, we determined the effect of Rbfox2 depletion on cell cycle progression by flow cytometry. The knockdown efficiency of siRbfox2 was confirmed by immunoblot analysis using anti-Rbfox2 antibody (Supplementary Fig. S1). Among cells transfected with control siRNA, there was no difference in the percentage of cells distributed in each stage of the cell cycle between untreated and arsenite-treated cells (Fig. 2a). Arsenite, which is known to induce oxidative stress by generating reactive oxygen species, was used as a stress inducer^28–30^. Unexpectedly, Rbfox2 depletion decreased the percentage of G1-phase cells from 63.4 to 52.1% in the absence of cellular stress, although cells shifted into the S and G2/M phases at equal rates. This finding suggested that Rbfox2 may be involved in cell cycle progression independent of a stress response and that its action is most likely in the nucleus. Notably, arsenite treatment induced an 8% decrease in the number of G1-phase cells and a 6% increase in the number of S-phase cells among Rbfox2-depleted cells. Because RB1 functions as a repressor of cell cycle progression from the G1 phase to the S phase, this stress-induced acceleration of G1- to S-phase progression in Rbfox2-depleted cells might be caused by reduced RB1 protein levels. We then examined whether alteration of cell cycle progression by Rbfox2 depletion under stress conditions exerts effects on cell growth. The results indicated that cellular stress significantly accelerated the growth of Rbfox2-depleted cells (Fig. 2b, *blue line*). Interestingly, despite changes in cell distribution among cell cycle stages (Fig. 2a), Rbfox2-depleted cells showed growth patterns similar to those of control cells in the absence of cellular stress (Fig. 2b), indicating that Rbfox2 does not significantly affect cell growth under normal conditions. These results strongly suggested that Rbfox2 participated in cell cycle regulation and cell proliferation by regulating RB1 protein levels under stress conditions.

### RB1 expression is significantly attenuated in colon cancer tissues

**Fig. 3:**
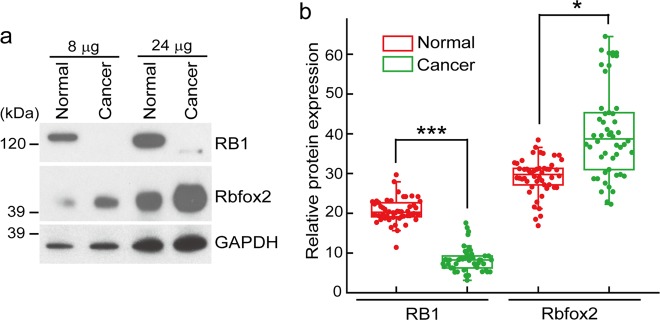
RB1 expression is markedly reduced in colon cancer tissues. **a** Immunoblot analysis of RB1 and Rbfox2 protein levels in noncancerous human colon and colon cancer tissues. Human tissue lysates (8 or 24 μg) were analyzed by SDS-PAGE/immunoblot analysis using anti-RB1 or anti-Rbfox2. The GAPDH level served as a loading control. **b** RPMA of RB1 and Rbfox2 protein levels in noncancerous colon (Normal) or colon cancer (Cancer) tissue (*n* = 50 per group). The scatter diagrams show the values for the individual samples in each group. The mean RB1- or Rbfox2-specific signal was quantified and is plotted. The data are presented as the mean ± SEM. *p < 0.0254 and ***p < 0.0005 compared to normal tissue

We have previously shown that RB1 expression is regulated by Rbfox2 under cellular stress conditions at the post-transcriptional level^22^. To study the role of Rbfox2 in colon cancer tissues through RB1 regulation, RB1 protein expression patterns were examined in normal and human colon cancer tissues by immunoblot analysis. We confirmed that Rbfox2 expression in cancer tissue was significantly elevated relative to that in normal tissue, whereas RB1 expression was markedly decreased in cancer tissue compared to normal tissue (Fig. 3a). To expand upon this observation, RPMA analysis of 50 samples of human colon cancer was performed using anti-RB1 and anti-Rbfox2 antibodies. Most colon cancer tissues displayed low RB1 protein levels relative to normal colon tissues, whereas Rbfox2 levels in colon cancer tissue were significantly higher than those in normal colon tissue (Fig. 3b). These results suggested Rbfox2 involvement in regulating the cell cycle and cell proliferation through regulation of RB1 protein levels in colon cancer tissue.

### Resveratrol inhibits SG-mediated Rbfox2 localization

**Fig. 4:**
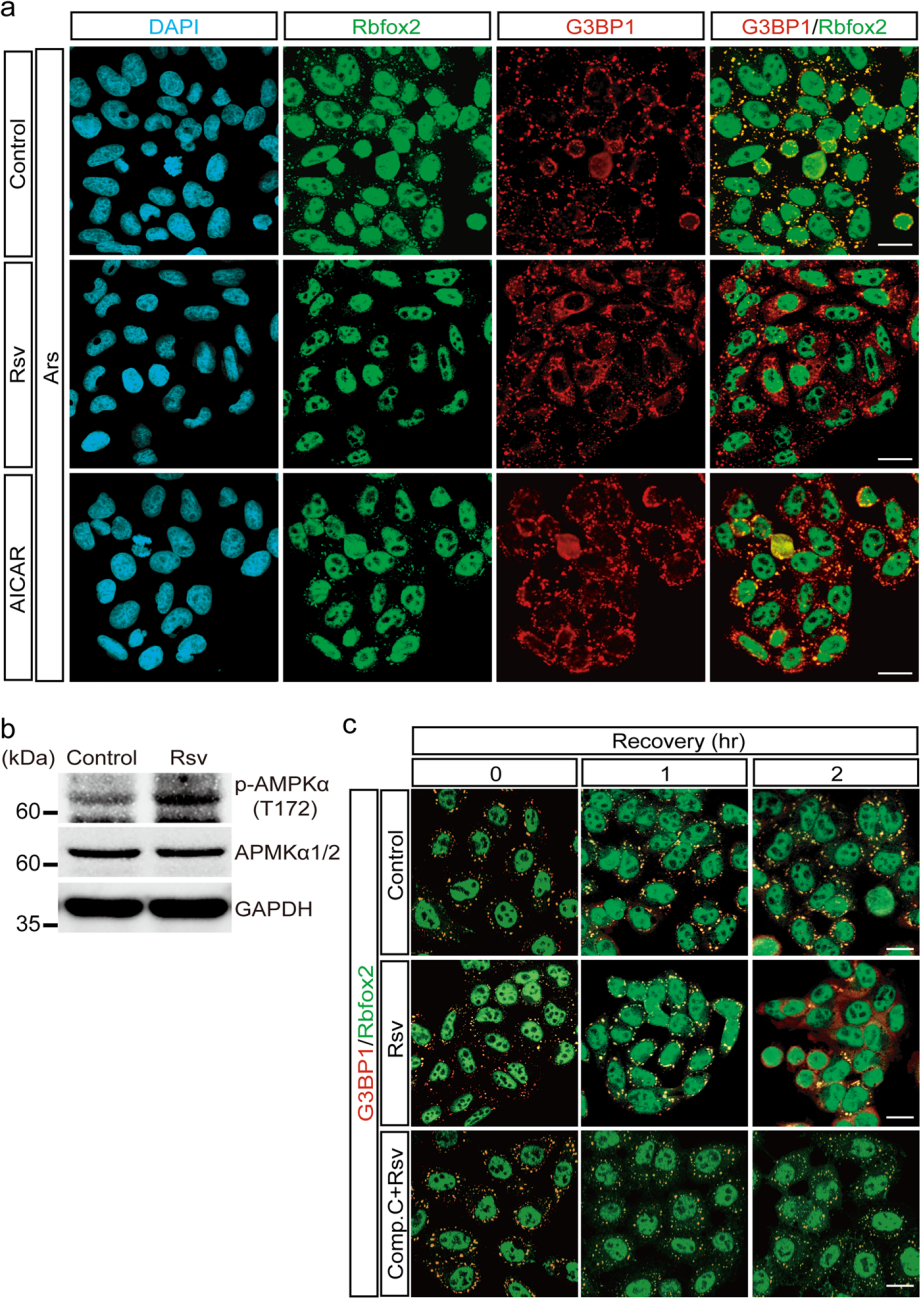
Resveratrol modulates Rbfox2 recruitment to SGs caused by arsenite treatment. **a** Immunofluorescence images of Rbfox2 (green) and G3BP1 (red) protein in arsenite-treated (100 μM; 40 min) HeLa cells (Control), in HeLa cells cotreated with arsenite after a 1-h pretreatment with 250 μM resveratrol (Rsv), and in HeLa cells cotreated with arsenite after a 12-h pretreatment with 500 μM AICAR (AICAR). DAPI was used to stain the nuclei. Scale bar, 20 μm. **b** HeLa cells were treated with 250 μM resveratrol for 1 h 40 min (Rsv). Immunoblot analysis was performed using anti-phospho-AMPKα (T172) and anti-AMPKα1/2 antibodies. GAPDH served as a loading control. **c** HeLa cells were treated with 100 μM arsenite for 40 min, and the 0-h time point was obtained at the end of treatment. For recovery, cells were incubated for 1 or 2 h in complete medium (Control) or medium containing 250 μM resveratrol (Rsv). Other cells were incubated for the same amount of time in medium containing 250 μM resveratrol and 10 μM compound C (Comp. C + Rsv). The fixed and permeabilized cells were coimmunostained with anti-Rbfox2 (green) and anti-G3BP1 (red). Scale bar, 20 μm

Our results showed that arsenite treatment induced the formation of Rbfox2-containing SGs that were coimmunostained with the SG marker G3BP1 (Fig. 4a). Antioxidants decrease the levels of reactive oxygen species to relieve oxidative stress^31,32^. Our observation that Rbfox2 is involved in cell cycle progression through regulation of RB1 levels in SGs prompted us to investigate whether an antioxidant dissociates Rbfox2 from SGs. Treatment with resveratrol (trans-3,5,4′-trihydroxystilbene), a well-known strong antioxidant^33^, significantly decreased arsenite-induced Rbfox2 SG localization (Fig. 4a). We confirmed the inhibitory effect of antioxidants on SG localization of Rbfox2 with N-acetyl cysteine (NAC), but NAC showed a weaker inhibitory effect than resveratrol (data not shown). These results suggest that resveratrol-mediated regulation of the SG localization of Rbfox2 might occur through antioxidant activity as well as through other types of activity. Interestingly, there was no clear difference in the SG localization of G3BP1 with resveratrol treatment. This result suggested that resveratrol specifically dissociated Rbfox2 from SGs.

Previous reports have demonstrated that resveratrol increases the levels of activated (phosphorylated) AMP-activated protein kinase (AMPK) in vivo, suggesting that AMPK might play a role in mediating resveratrol-specific effects^34,35^. We confirmed the effect of resveratrol on Thr172 phosphorylation of AMPK (Fig. 4b). To elucidate the role of AMPK in the SG localization of Rbfox2, we treated cells with AICAR, an AMP mimetic that directly activates AMPK. AMPK phosphorylation by AICAR had an effect on the SG localization of Rbfox2 similar to that observed following resveratrol treatment (Fig. 4a), demonstrating that AMPK is a key molecule involved in the resveratrol-mediated SG localization of Rbfox2. Moreover, the intracellular localization of Rbfox2 and G3BP1 was not altered by treatment with either resveratrol or AICAR (Supplementary Fig. S2a). Interestingly, although treatment with an AMPK inhibitor (compound C) did not induce SG formation, it did induce Rbfox2 translocation from the nucleus to the cytoplasm (Supplementary Fig. S2b). We then examined the effect of resveratrol on the dissociation of Rbfox2-containing SGs during alleviation of cellular stress. We found that SGs were undetectable after a 2-h recovery period in the presence of resveratrol, whereas SGs were still observed in the absence of resveratrol (Fig. 4c). To investigate the role of resveratrol on AMPK, cells were cotreated with compound C and resveratrol during recovery. Compound C treatment efficiently inhibited the effects of resveratrol on the SG localization of Rbfox2 (Fig. 4c). These results suggested that SG-mediated Rbfox2 localization could be modulated by resveratrol treatment via the AMPK pathway.

### Resveratrol regulates the posttranslational modification (PTM) of Rbfox2

**Fig. 5:**
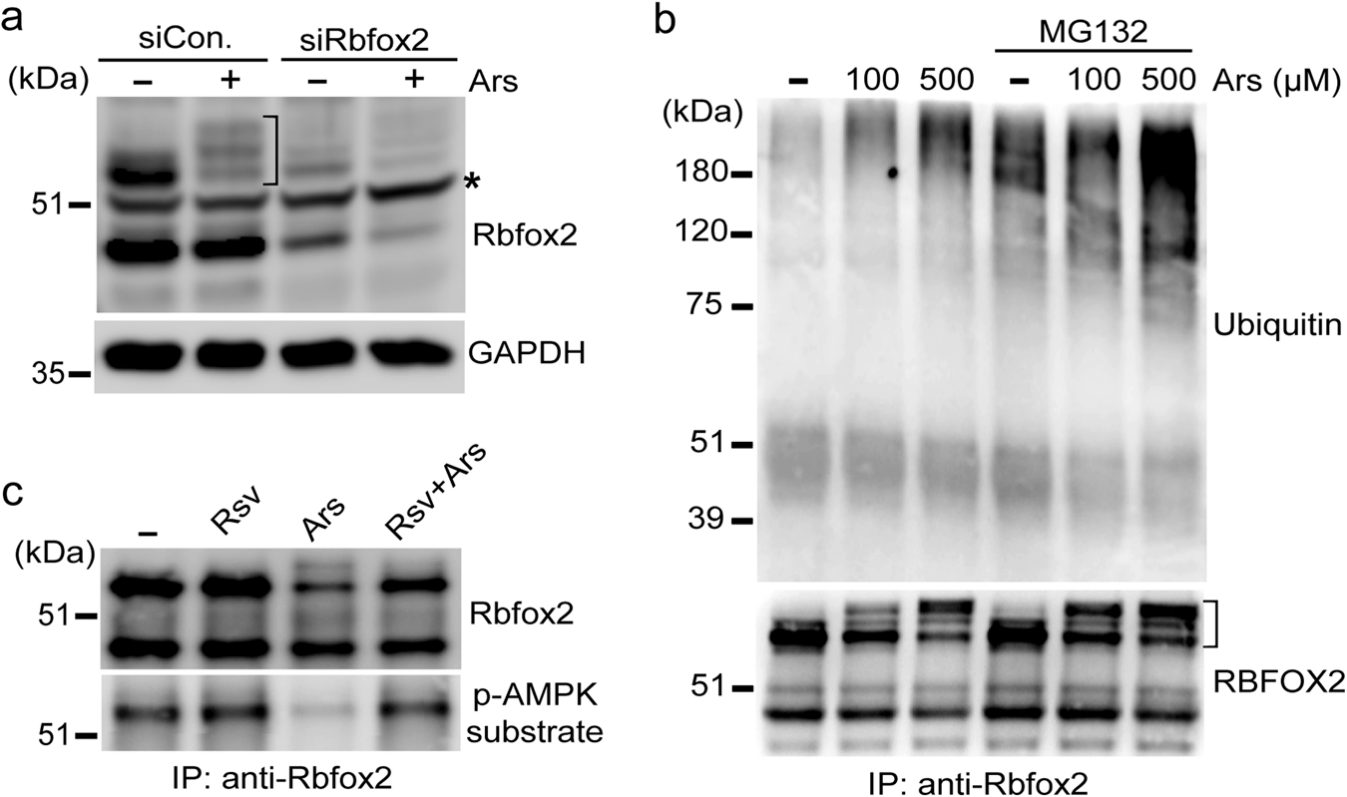
Arsenite treatment induces PTM of Rbfox2. **a** HeLa cells were transfected with siRbfox2 or siControl (siCon.). After 36 h of incubation, the cells were treated with 500 μM sodium arsenite for 40 min. The cell extracts were subjected to immunoblot analysis with anti-Rbfox2 antibody. GAPDH served as a loading control. Nonspecific bands are indicated with asterisks. **b** HeLa cells were treated with 50 μM MG132 for 90 min before 100 µM or 500 µM arsenite treatment for 40 min. The cell lysates were immunoprecipitated with anti-Rbfox2 antibody. Immunoblot analysis was performed using anti-ubiquitin and anti-Rbfox2 antibodies. **c** HeLa cells were treated with 250 μM resveratrol for 1 h 40 min (Rsv) or 100 μM arsenite for 40 min (Ars). Other HeLa cells were pretreated with 250 μM resveratrol for 1 h and then cotreated with 100 μM arsenate for 40 min in the presence of resveratrol (Rsv + Ars). Cell lysates were prepared and immunoprecipitated with anti-Rbfox2 antibody, and immunoblot analysis was performed

Next, we investigated whether the PTM of Rbfox2 is altered under stress conditions. To confirm the specific Rbfox2 protein band, knockdown of Rbfox2 using siRbfox2 was performed. Arsenite treatment not only reduced the expression level of Rbfox2 but also shifted the protein band upwards (Fig. 5a). Through the knockdown of Rbfox2 by siRbfox2, we confirmed that the shifted protein was indeed Rbfox2 protein. We next questioned whether the shift and decrease in Rbfox2 protein expression by arsenite treatment was due to its ubiquitination. We monitored changes in the ubiquitination status of Rbfox2 after arsenite treatment in the presence of the proteasome inhibitor MG132. As shown in Fig. 5b, arsenite treatment substantially increased Rbfox2 ubiquitination. Moreover, MG132 treatment markedly shifted the Rbfox2 protein band upwards and increased the ubiquitination of the Rbfox2 protein. Our observation that activation of AMPK by resveratrol inhibits the SG localization of Rbfox2 prompted us to examine the changes in the phosphorylation status of Rbfox2 under stress conditions. Phosphorylation of Rbfox2 was tested using a phospho-(Ser/Thr) AMPK substrate antibody after arsenite treatment. Arsenite treatment decreased the phosphorylation of Rbfox2, whereas resveratrol treatment restored the phosphorylation of Rbfox2 (Fig. 5c). These results suggest that changes in PTMs, including ubiquitination and phosphorylation of the Rbfox2 protein, occur under stress conditions and could mediate the changes in the phosphorylation of the Rbfox2 protein caused by treatment with resveratrol.

### Resveratrol inhibits cancer progression

To investigate the effect of resveratrol-mediated suppression of SG-mediated Rbfox2 localization on cancer progression, we used a B16-F10 murine melanoma model. B16-F10 cells show different tumor formation ability depending on the injection method in mice. Therefore, this model system was used to confirm various effects of resveratrol.

**Fig. 6:**
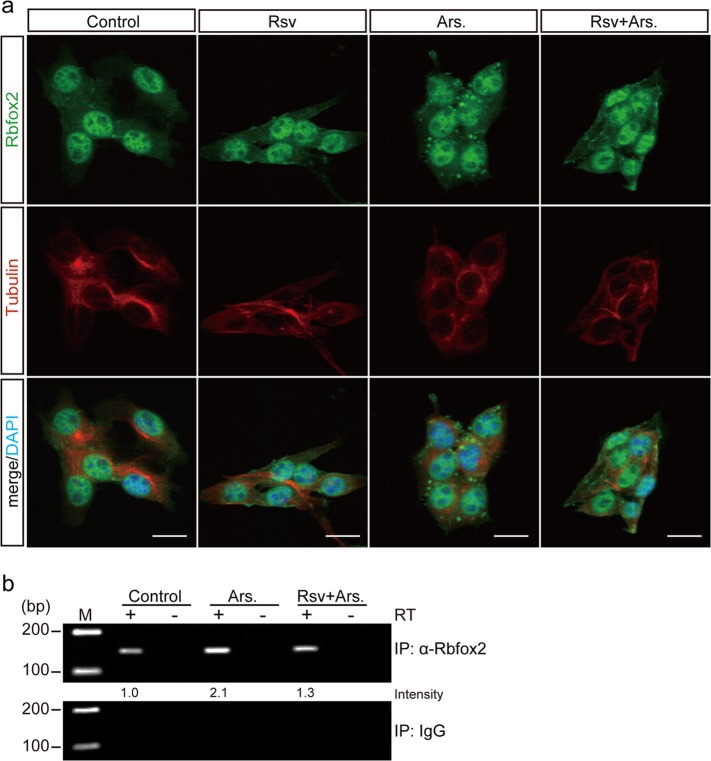
Resveratrol inhibits Rbfox2 localization to SGs in B16-F10 cells. **a** B16-F10 cells were treated with 250 μM resveratrol for 5 h (Rsv) or 500 μM arsenite for 1 h (Ars.). Other B16-F10 cells were pretreated with 250 μM resveratrol for 4 h and then cotreated with 500 μM arsenate for 1 h in the presence of resveratrol (Rsv + Ars.). The fixed and permeabilized cells were coimmunostained with anti-Rbfox2 (green) and anti-tubulin (red). Scale bar, 20 μm. **b** B16-F10 cells were treated with 500 μM arsenite for 1 h (Ars.) or cotreated with arsenite after 4 h of pretreatment with 250 μM resveratrol (Rsv + Ars.). RIP was performed using anti-Rbfox2, and then RT-PCR was performed using *RB1* mRNA primers in B16-F10 cells. IgG was used as a negative control. RT + indicates the presence of reverse transcriptase during cDNA synthesis. RT- indicates control lanes including samples with no reverse transcriptase to verify the absence of genomic DNA contamination. The values under the gels denote the relative intensities of the cDNA bands. M, marker

First, we examined SG-mediated Rbfox2 localization, finding that SG formation decreased following resveratrol treatment in B16-F10 cells (Fig. 6a). Additionally, we confirmed increases in Rbfox2 binding to *RB1* mRNA following arsenite treatment (Fig. 6b) that were subsequently abolished by resveratrol treatment, confirming that resveratrol specifically inhibited SG-mediated Rbfox2 localization.

**Fig. 7:**
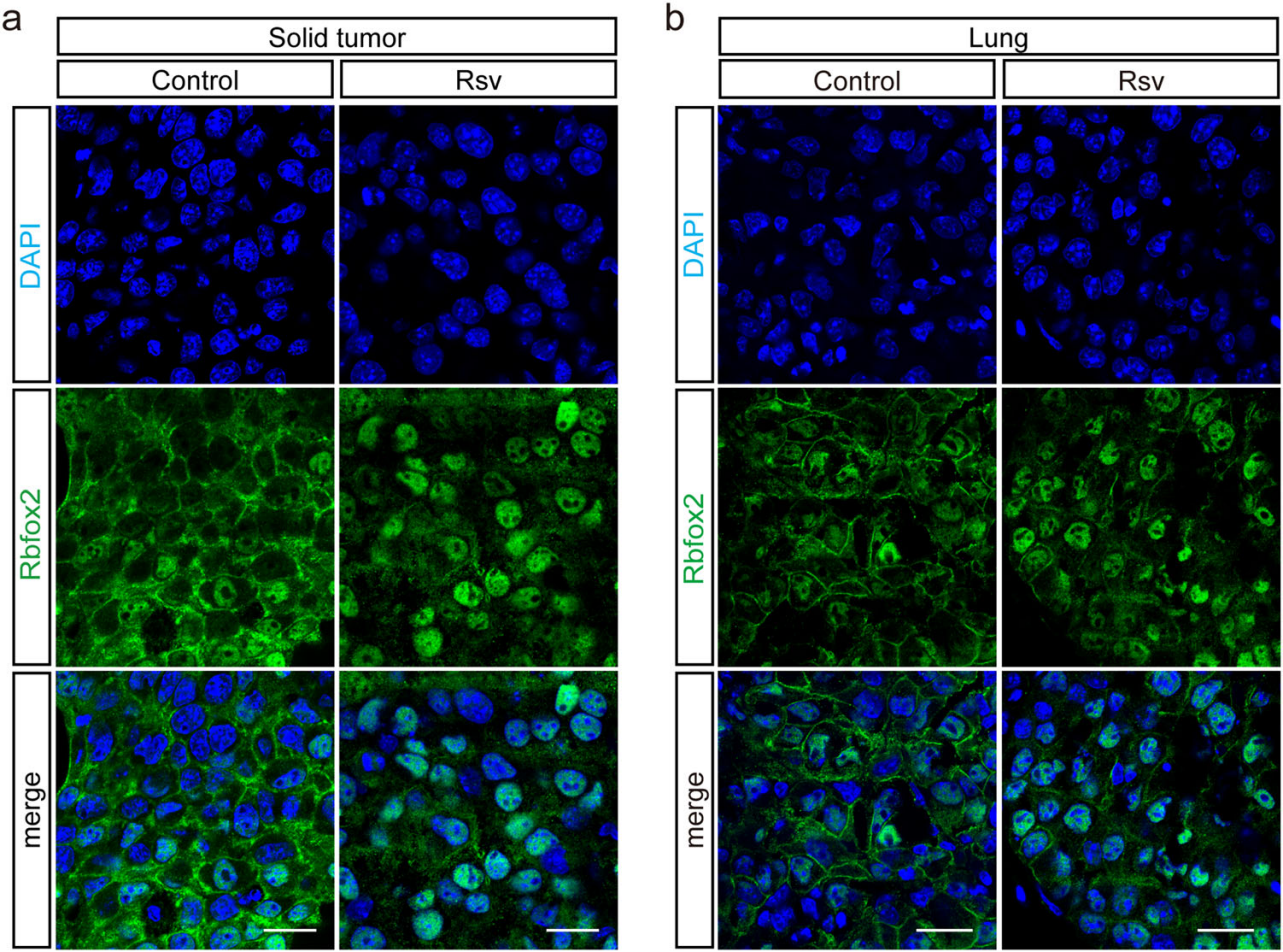
Resveratrol treatment enhances the nuclear localization of Rbfox2 in a primary tumor model of B16-F10 cells. **a** B16-F10 cells (5 × 10^5^) were injected subcutaneously into C57BL/6 J mice (Solid tumor). Mice were IP injected with 1 mg/kg resveratrol (Rsv) or vehicle (Control) once daily beginning on the day prior to the injection of B16-F10 cells. Six days after the injection of the cells, the mice were sacrificed, and tumors were isolated. Immunofluorescence images of Rbfox2 protein (green) in the tumor samples were produced. The nuclei were stained with DAPI (blue). Scale bar, 20 μm. **b** Six-week-old male C57BL/6 J mice were intravenously injected in the tail vein with B16-F10 cells (2 × 10^5^, Lung). The mice were administered 1 mg/kg resveratrol (Rsv) or vehicle (Control) through IP injection once a day beginning on the day before injection of B16-F10 cells. Fourteen days after cell injection, the mice were sacrificed, and their lungs were isolated. Immunofluorescence images of Rbfox2 (green) and tubulin (red) protein in mouse lung tissues are shown. The nuclei were stained with DAPI (blue). Scale bar, 20 μm

Next, we investigated the anticancer effect of resveratrol on B16-F10 melanoma cells. B16-F10 cells were subcutaneously injected into mice, which then received resveratrol administration (1 mg/kg; IP) daily. Six days post injection, the mice were sacrificed, and the solid tumors were prepared for immunofluorescence microscopy. Although Rbfox2 showed cytoplasmic staining in tissues from mice without resveratrol treatment, resveratrol treatment enhanced the nuclear localization of Rbfox2 (Fig. 7a). Furthermore, resveratrol treatment significantly reduced tumor size (Supplementary Fig. S3a). This observation was confirmed by statistical analysis of tumor volume, which confirmed that there were significant differences in the resveratrol-treated group relative to the untreated group (Supplementary Fig. S3b). To confirm this observation, B16-F10 cells were injected into the lateral tail vein of mice, and the mice were then treated daily with resveratrol. At 14 days post injection, the lungs were isolated from the mice and prepared for immunofluorescence microscopy to examine the intracellular localization of Rbfox2 (Fig. 7b). We confirmed that resveratrol treatment enhanced the nuclear localization of Rbfox2. In addition, 14 days of resveratrol treatment significantly reduced the number of nodules per mouse (Supplementary Fig. S3c and d). These results indicated that resveratrol exerted anticancer effects in vivo.

## Discussion

In this study, we investigated the anticancer efficacy of Rbfox2 dissociation from *RB1* mRNA in SGs. Immunofluorescence microscopy revealed that Rbfox2 exhibited nuclear localization in normal tissue but exhibited cytoplasmic localization in human colon cancer tissue. Additionally, we observed significantly lower RB1 protein levels in human colon cancer tissues than in normal tissues and found that Rbfox2 plays an important role in cell cycle progression, regardless of cellular stress. Moreover, Rbfox2 reduced RB1 protein levels under stress conditions and promoted cancer cell cycle progression and proliferation. Furthermore, the SG localization of Rbfox2 was inhibited by resveratrol treatment through PTM of Rbfox2, resulting in rapid SG dissociation during recovery. Similarly, in a mouse primary tumor model, Rbfox2 localization and cancer proliferation were inhibited by resveratrol administration.

The Rbfox2 gene is known to produce several splicing isoforms, and its isoforms exhibit different subcellular localization^36^. In our previous study, we showed that endogenous Rbfox2 isoforms, which are predominantly localized in nuclei, are localized in SGs in the cytoplasm under stress conditions^22^. Under normal conditions, the amount of Rbfox2 expressed in the cytoplasm is significantly lower than that expressed in nuclei, but under stress conditions, Rbfox2 translocates from the nucleus into the cytoplasm. Cytoplasmic isoform(s) of Rbfox2, which seems to be diffusely distributed in unstressed cells, might also be recruited to SGs under stress conditions. When Rbfox2 is present in the cytoplasm, stress granule formation is faster and stronger than when Rbfox2 is present in the nucleus, enabling the granules to be assembled more quickly under stress conditions. SGs are also difficult to observe in the form of granules in vivo, unlike in vitro, because assembly and disassembly occur rapidly in stressful environments. However, we were able to confirm the cytoplasmic localization of Rbfox2 in vivo in this study, which means that Rbfox2, which functions as an alternative splicing regulator in the nucleus, performs novel functions outside of the nucleus.

Rbfox2 is uniformly expressed in tissues from the developmental to adult stages and is involved in splicing regulation in the nucleus. Here we demonstrated that Rbfox2 migrates to the cytoplasm in response to external stress and modulates the expression of target mRNAs associated with cancer development, thereby affecting cancer progression. The RNA recognition motif domain of Rbfox-family proteins binds to the 3′ UTR region of target mRNAs and contributes to mRNA stabilization^37–39^. A potential cause of various diseases involves binding of RBPs (including Rbfox2) to disease-associated mRNAs in SGs to inhibit gene expression. Rbfox2 levels were elevated in colon cancer tissue relative to normal colon tissue, whereas RB1 protein levels were lower in cancer tissue. Upon resolution of cellular stress conditions, SGs dissociate; however, in cells damaged to a degree that results in difficulty recovering due to continuous external stimulation, SGs are presumably maintained and can support the development of cells into cancer cells. Therefore, regulation of the interaction of Rbfox2 with SGs promotes target mRNA dissociation from SGs and normalization of cell function. In this study, we showed that in vitro and in vivo treatment with resveratrol, a plant-derived natural compound, regulated the interaction of Rbfox2 with SGs, thereby demonstrating that Rbfox2 is a potential target for the development of new anticancer drugs.

Rbfox2 is involved in mesenchymal tissue-specific splicing during the epithelial-mesenchymal transition, which is important for cancer cell metastasis^40^. The Rbfox2 binding site is involved in alternative splicing events associated with cancer-related genes in breast and ovarian cancers^41^. In the present study, we confirmed that Rbfox2 affected cell cycle progression, regardless of stress conditions. This was presumably a consequence of Rbfox2 acting as an alternative splicing regulator of cell cycle control genes. Additionally, our findings demonstrated, for the first time, that Rbfox2 regulated target RNA expression in the cytoplasm in addition to regulating splicing in the nucleus and thereby suggest Rbfox2 to be an important mediator of disease/cancer progression. Furthermore, the in vivo mouse model demonstrated that resveratrol administration inhibited Rbfox2 localization to the cytoplasm and inhibited cancer progression.

Resveratrol is a phytoalexin synthesized by plants in response to pathogen infection and belongs to the polyphenol group. As a powerful antioxidant, resveratrol has been used to study various diseases, including cancer, based on its naturally occurring compounds. Previous studies have reported that resveratrol has an anticancer effect against tumor initiation and cancer progression. In particular, activation of AMPK by resveratrol inhibits both cyclooxygenase 2 activity, which affects tumor formation, and mechanistic target of rapamycin signaling^42–44^. Additionally, resveratrol treatment reduces the DNA-binding capacity of nuclear factor-κB, a transcription factor necessary for oncogene transcription^45–48^. Moreover, resveratrol treatment induces tumor cell apoptosis and cell cycle arrest; however, the relationship between resveratrol and SGs has not been extensively investigated. In the present study, we proposed a novel resveratrol-specific mechanism associated with various pathways described previously. Our results indicated the normalizing effect of resveratrol on the transcription and translation of cell cycle regulatory genes in association with Rbfox2 dissociation from SGs. Specifically, upon AMPK activation, resveratrol stimulated the formation of SGs by increasing stress-induced eIF2α phosphorylation^49,50^. This effect likely promoted SG formation and effected changes in RBP composition in SGs that altered cell survival, cell signaling, and translation initiation. Furthermore, resveratrol specifically restored oxidative stress-induced PTMs of Rbfox2, suggesting that the PTM status of Rbfox2 is important for the SG localization of Rbfox2.

In addition to serving as mRNA storage sites, SGs also serve as flexible sites of gene expression and as signaling hubs that affect multiple aspects of cellular metabolism^7^. Because SG assembly and disassembly in the cytoplasm affect cell signaling and survival, studies on these mechanisms can be essential for elucidating disease pathology and can be important for identifying targets for disease treatment. Cancer causes hyperosmolarity, hypoxia, and nutrient depletion due to a lack of oxygen and nutrients resulting from rapid tumor growth despite new blood vessel formation^51^. Additionally, inappropriate protein folding and synthesis lead to endoplasmic reticulum stress and the generation of reactive oxygen species, which increase the exposure of cancer cells to various stressors^52^. To date, studies on intracellular stress and cancer have focused on controlling protein folding through activity involving heat-shock proteins and through prevention of nonspecific protein aggregation^53^. The present study focused on the involvement of SGs in cancer initiation and progression in response to intracellular stress, a novel aspect of cancer progression, and offers further insight into the development of new cancer-related therapeutics.

## Supplementary information

Supplementary Information
