# Supplementary material for: Rbfox2 dissociation from stress granules suppresses cancer progression
Source: Exp Mol Med. 2019 Apr 26;51(4):49. doi: 10.1038/s12276-019-0246-y (PMC6486603; doi:10.1038/s12276-019-0246-y)
Supplement: Supplementary file 1 — Supplementary Information [file 12276_2019_246_MOESM1_ESM.docx]

**Supplementary Figure and Legend**


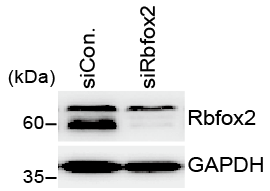


**Fig. S1 Specific knockdown of Rbfox2 expression by the siRbfox2.** HeLa cells were transfected with siControl (siCon.) or siRbfox2 for 36 h. Representative immunoblot showing the effects of siRNA transfections on protein levels of Rbfox2. GAPDH served as loading control.


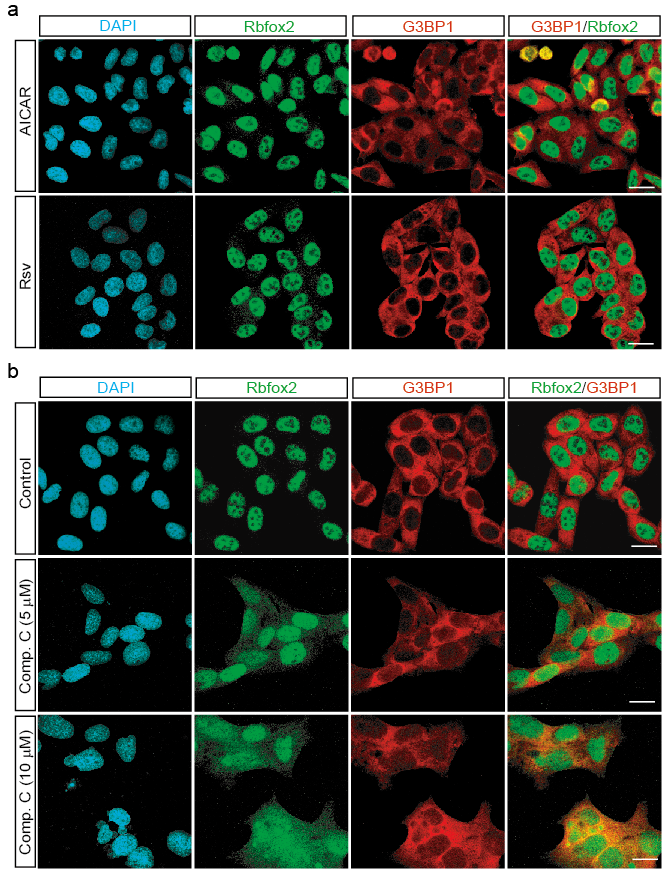


**Fig. S2 Resveratrol, AICAR, or Compound C treatment of HeLa cells does not induce SG formation. a** Immunofluorescence images of Rbfox2 (green) and G3BP1 (red) proteins visualized in HeLa cells treated with 500 μM AICAR for 12 h and 40 min (AICAR). Immunofluorescence images of Rbfox2 and G3BP1 proteins visualized in HeLa cells treated with 250 μM resveratrol for 1 h and 40 min (Rsv). DAPI staining represents nuclei. Scale bar, 20 μm. **b** Immunofluorescence images of Rbfox2 (green) and G3BP1 (red) proteins visualized in HeLa cells treated with 5 μM or 10 μM compound C for 12 h (Comp. C). DAPI staining represents nuclei. Scale bar, 20 μm.


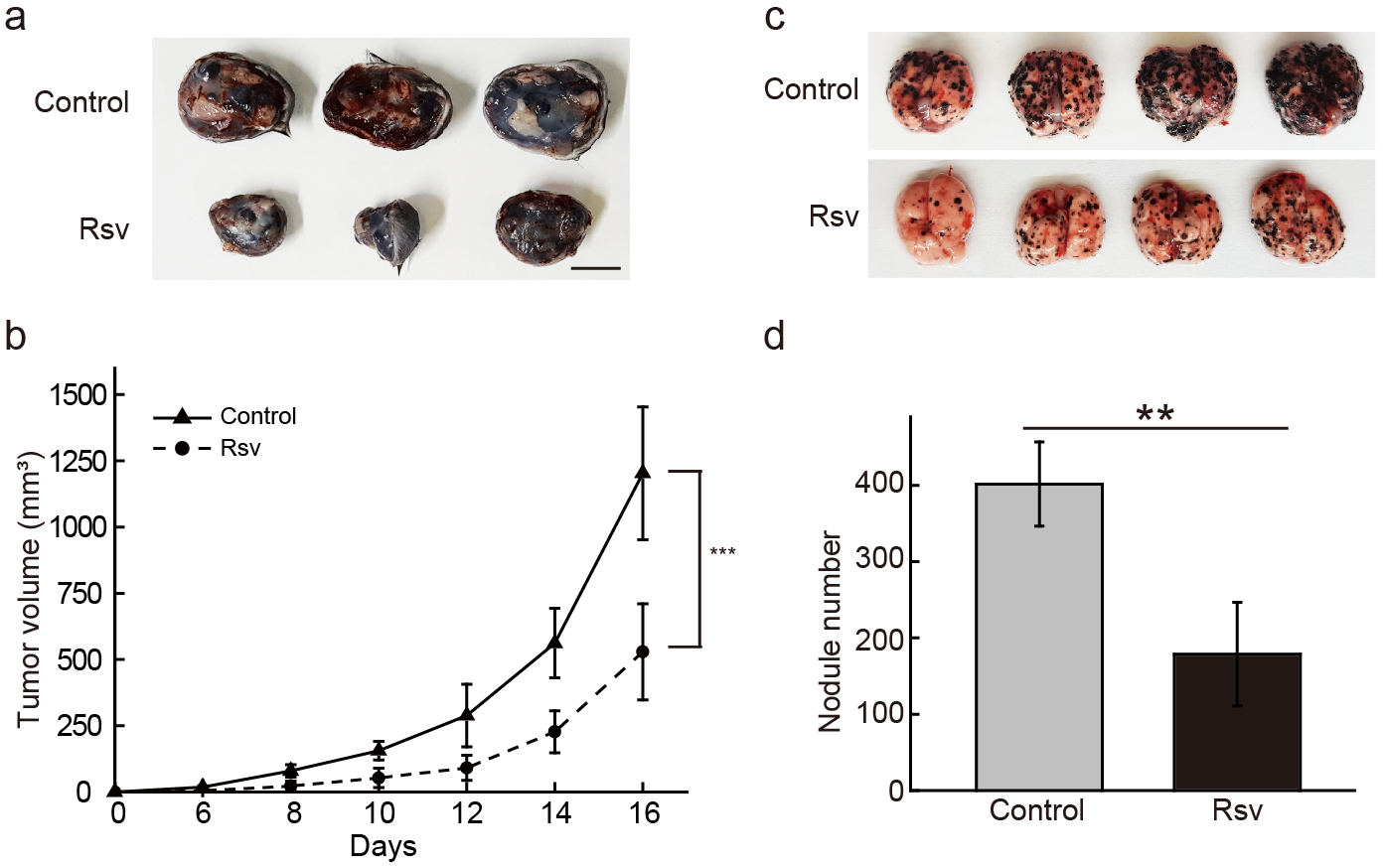


**Fig. S3 Resveratrol inhibits cancer progression.** **a** Representative image of solid tumors from the control and resveratrol treatment groups (Rsv) produced 16 days after the subcutaneous injection of B16-F10 cells. Scale bar, 10 mm. **b** Growth curves based on the solid tumor volumes were monitored every two days after implantation. Data are presented as the mean ± SEM; n = 6 per group. ****p* < 0.001 vs. Control. **c** Representative photograph of lung metastatic nodules produced 14 days after intravenous injection of B16-F10 cells. **d** The number of lung metastatic nodules were counted and represented as the mean ± SD of each group (*n* = 4/each). ***p* < 0.005 vs. Control.
